# Emergency room surgical workload in an inner city UK teaching hospital

**DOI:** 10.1186/1749-7922-3-19

**Published:** 2008-05-30

**Authors:** Tuong A Mai-Phan, Bijendra Patel, Michael Walsh, Ajit T Abraham, Hemant M Kocher

**Affiliations:** 1 Institute of Cancer, Barts and the London School of Medicine and Dentistry, London, UK; 2 The Department of General Surgery, The Royal London Hospital, Barts and the London NHS Trust, Whitechapel, London E1 1BB, UK

## Abstract

**Background:**

Emergency admissions may account for over 50% of surgical admissions. The impact on service provision and implications for training are difficult to quantify. We performed a cohort study to analyse these workload patterns.

**Methods:**

Data on emergency room (ER) surgical admissions over six months was collected including patient demographics, referral sources, diagnosis, operation and length of stay and analysed according to sub-speciality and age-groups.

**Results:**

There were 1392 (median age 41 (IQR 28–60) years, M:F = 1.7:1) emergency surgical admissions over six months; 45% were under 40 years of age and 48% patients self-referred to the ER. The commonest diagnoses were abscesses (11%), non-specific abdominal pain (9.7%) and neuro-trauma (9.6%). The median length of stay was 4 (IQR 2–8) days; with older (>80 years) patient staying significantly longer than those <40 years of age (median 8 vs 2 two days, P < 0.0001, Kruskal-Wallis test). Vascular patients remained in hospital longer than trauma or general surgery patients (median 14 vs 3 days, P < 0.0001, Kruskal-Wallis test). A high proportion (43.5%) of the patients required operative intervention and service implications of various diagnoses and operative interventions are highlighted.

**Conclusion:**

With the introduction of shortened training period in Europe and World over, trainees may benefit from increased exposure to trauma and surgical emergencies. Resource planning should be based on more comprehensive, prospective data such as these.

## Introduction

Emergency surgical admissions account for 46% to 57% of all surgical admissions [1-3] but workload estimates are difficult to achieve because of the unpredictability and variability of such admissions. There are no contemporaneous studies concerning the nature and volume of emergency surgical admissions. The impact of the emergency surgical workload on surgical practice is not only determined by overall volume but also by patient demographics, appropriateness of referral, centralisation, diagnoses, and required surgical operations. [4] The changing patterns have implications for surgical training, workforce planning and service provision. [2] The Royal London Hospital, a multi-specialty inner city teaching hospital which provides London's only Air Ambulance caters to a young, ethnically & socio-economically diverse, mainly immigrant population. [5] Health services in London are to be reconfigured, with fewer centres catering to larger populations and this similar exercise is being carried out in different parts of the world for macro- and micro-economic reasons without adequate data on volume, length of stay and problems for various specialties in hospitals. [6] This study sought to identify the current patterns and common problems related to emergency room (ER) admissions from a single hospital.

## Methods

All ER surgical admissions over six months (12 January – 11 July 2007) to accident and emergency department were recorded prospectively. Orthopaedic trauma only (not polytrauma) and urological admissions were excluded since they were managed by orthopaedic and urology departments respectively; patients referred internally (already in-patient for another medical condition) from other specialties were also excluded since they did not effect the surgical department's bed occupancy rates. Information was obtained from hand-over lists and the Electronic Patient Record (EPR) viewer, an intranet-based patient record of Barts and The London NHS Trust. All data were anonymised and recorded in password protected spreadsheets. Information regarding time of operation was extracted from theatre logs. The final diagnosis was determined after investigations and/or operation and all patients were followed-up till discharge. One overnight stay was classified as 1 day of stay for length of stay calculations. Statistical analysis (ANOVA and Kruskal-Wallis test for data with and without normal distribution respectively) were performed on SPSS 14.0 for Windows (SPSS UK Ltd, Surrey, UK).

## Results

**Figure 1:**
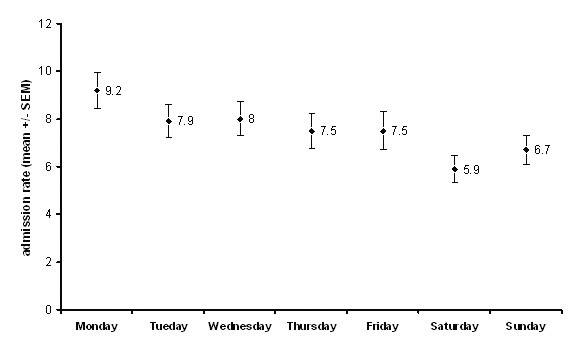
Admissions per day of the week. Results are expressed as mean ± standard error of mean.

**Figure 2:**
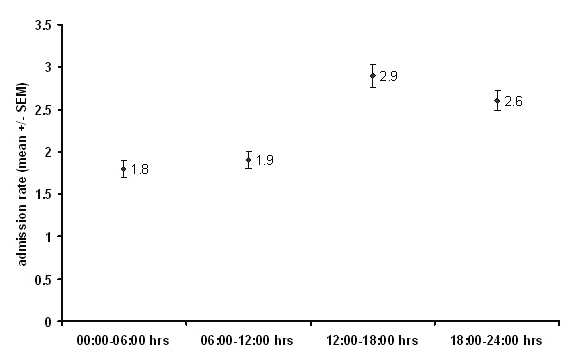
Timing of admission and peak hours. Results are expressed as mean ± standard error of mean.

**Figure 3:**
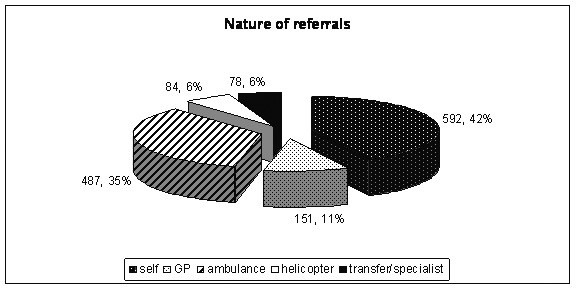
Nature of referrals.

**Figure 4:**
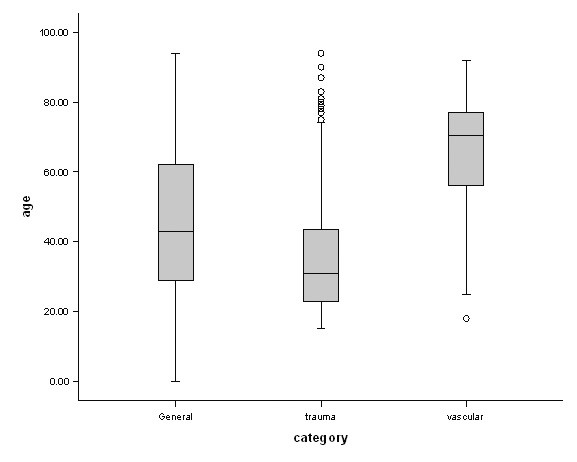
Median age per specialty distribution.

1775 emergency surgery referrals were recorded, of which 1392 (870 (62%) male, median age 41 (Inter-quartile range (IQR) 28–60 years) were admitted for further treatment over a six month period resulting in a mean of 7.2 admissions per day, with Saturdays being the days with least admissions (ANOVA, df = 6, F = 2.149, P = 0.05; Figure 1). Forty five per cent (402) admissions were under 40 years of age. The busiest time of day for admission was between 12.00 and 18.00 hours when (36%) were admitted (ANOVA, df = 3, F = 24.42, P < 0.001, Figure 2). Interestingly GP (General Practitioner) referrals (those patients vetted initially by family physicians) formed only 11% of all emergency surgical admissions (Figure 3). Trauma patients were significantly younger than vascular patients (Kruskal-Wallis, df = 2, Chi-square statistic = 196.2, P < 0.001; Figure 4).

### Diagnoses & Operations

Table 1 details the distribution of patients according to specialty, diagnosis, number of admissions as well as sex ratios, and length of stay. Abscesses (11%), non-specific abdominal pain (9.7%) and acute appendicitis (6.6%) were the commonest general surgical diagnoses. Among trauma patients neurological injury (9.6%) was the commonest reason for admission. As expected intuitively, male to female ratio was different according to specialties: vascular (2.8:1), trauma (4.4:1), general surgery (1.1:1). The younger patients (<40 years) had a significant male preponderance (1.8:1) and the sex ratio became 1 only in over 80 year age group. Of all emergency surgical admissions, 605 (43.5%) required operative intervention. More than half (346 (57%)) of general surgical emergency admissions required operative intervention while only 32% (195) of trauma and 11% (64) of vascular emergencies required operative intervention. Types of operations, demographics, length of stay are detailed in table 2.

**Table 1:** Common diagnoses

**Diagnosis**	**N (%)**	**M**	**F**	**Median age (IQR)**	**Mean LOS (SEM)**	**Median LOS (IQR)**
**Trauma**	**387 (27.9)**	**315**	**72**	**31 (23–44)**	**9 (0.8)**	**3 (1–9)**
Neuro trauma	133 (9.6)	105	28	34 (23–47)	8 (1.23)	2 (1–9)
Thoracic trauma	45 (3.2)	40	5	36 (21–42)	10 (2.44)	3 (1–7)
Abdominal trauma	30 (2.2)	25	5	29 (22–44)	6 (1.38)	4 (1–8)
Orthopaedic trauma	123 (8.8)	98	25	30 (23–42)	8 (1)	3 (1–11)
Vascular trauma	3 (0.2)	2	1	32 (30–70)	4 (1.45)	4 (2–7)
Maxillo-facial trauma	21 (1.5)	18	3	24 (20–37)	3 (0.84)	2 (1–5)
Poly trauma	27 (1.9)	24	3	30 (22–44)	27 (6.18)	10 (4–52)
Others trauma	5 (0.4)	3	2	38 (29–39)	5 (2.53)	3 (0–10)
**Vascular**	**110 (7.9)**	**81**	**29**	**71 (56–77)**	**18 (1.42)**	**14 (8–28)**
Abdominal aortic aneurysm	25 (1.8)	21	4	77 (64–82)	12 (2.21)	10 (6–17)
Ischaemic foot	36 (2.6)	30	6	69 (55–75)	24 (2.27)	23 (12–30)
Embolic episodes	12 (0.9)	6	6	61(49–73)	16 (4.47)	9 (7–19)
Peripheral vascular diseases	25 (1.8)	17	8	75 (59–81)	16 (2.77)	15 (6–22)
Others	12 (0.9)	7	5	64 (34–74)	13 (3.68)	12 (6–20)
**General surgery**	**895 (64.2)**	**474**	**421**	**43 (29–62)**	**6 (0.28)**	**3 (2–7)**
Appendicitis	96 (6.9)	60	36	27 (22–36)	4 (0.43)	3 (2–5)
Abscess	153 (11.0)	98	55	38 (29–47)	3 (0.45)	2 (1–3)
Hernia	32 (2.3)	20	12	58 (37–67)	6 (1.73)	3 (1–5)
GDU perforation	18 (1.3)	8	**10**	59 (41–72)	10 (1.6)	9 (6–12)
Pancreatitis	55 (4.0)	30	25	50 (37–60)	9 (0.9)	4 (2–7)
Adhesion	8 (0.6)	4	4	29 (25–45)	5 (1.19)	5 (2–8)
Obstruction	26 (1.9)	16	10	67 (40–74)	11 (1.81)	7 (5–15)
Gallbladder disease	67 (4.8)	24	**43**	42(29–70)	8 (1.1)	4 (2–9)
Biliary tract disease	15 (1.1)	7	8	56 (41–63)	5 (0.87)	4 (2–6)
NSAP	135 (9.7)	63	**72**	37 (27–55)	5 (0.71)	3 (1–5)
Diverticular disease	24 (1.7)	7	**17**	54 (41–63)	5 (0.87)	4 (2–6)
UGI bleeding	5 (0.4)	3	2	54 (46–59)	15 (7.11)	11 (4–13)
LGI bleeding	24 (1.7)	14	10	64 (57–78)	5 (0.99)	3 (3–5)
Malignacy	31 (2.2)	13	**18**	61 (50–69)	12 (2.52)	9 (3–16)
Urology conditions	27 (1.9)	21	6	51 (31–67)	4 (1.57)	2 (1–5)
Gyneacological conditions	23 (1.7)	0	**23**	31 (21–43)	4 (1.23)	3 (1–5)
Medical conditions	73 (5.2)	36	**37**	51 (31–71)	10 (1.63)	5 (2–12)
Others	50 (3.6)	31	19	50 (37–72)	7 (0.94)	5 (2–9)
Post operative problems	24 (1.7)	16	8	50 (39–65)	7 (1.76)	3 (3–8)
Miscellaneous	9 (0.6)	3	**6**	54 (38–59)	6 (1.49)	6 (2–9)
**Overall**	**1392**	**870**	**522**	**41 (28–60)**	**8 (0.32)**	**4 (2–8)**

N: number. M: male. F: female. SEM: standard error of mean. IQR: interquatile range. GDU: gastroduodenal ulcer. NSAP: non-specific abdominal pain. UGI: upper intestinal. LGI: lower gastrointestinal.

**Table 2:** Emergency operations.

**Operation**	**N (%)**	**M**	**F**	**Median age (IQR)**	**Mean LOS (± SEM)**	**Median LOS (IQR)**
**Trauma**	**195 (32)**	**162**	**33**	**30 (21–42)**	**14.90 (1.54)**	**6 (3–16)**
Laparotomy	17 (2.8)	16	1	29 (22–37)	16.4 (7.74)	6 (4–9)
Thoracic procedures	33 (5.5)	29	4	29 (20–42)	6.9 (1.3)	5 (2–8)
Neuro surgery	30 (5)	23	7	35 (21–42)	24.2 (4.16)	16 (12–29)
Orthopaedic operations	52 (8.6)	41	11	31 (24–45)	16.8 (2.9)	10 (5–18)
Maxillo facial operations	6 (1)	4	2	18 (17–22)	8 (2.84)	5.5 (4–7)
Vascular operations	6 (1)	5	1	31 (25–32)	14.3 (9.98)	5.5 (2–7)
Minor surgery	36 (6)	34	2	29 (20–42)	2.8 (0.5)	2 (1–3)
Poly trauma	15 (2.5)	13	2	32 (24–44)	39.7 (7.97)	38 (8–65)
**Vascular**	**64 (11)**	**48**	**16**	**72 (62–77)**	**24.8 (2.65)**	**18.5 (10–31)**
AAA repair	7 (1.2)	6	1	77 (67–83)	22 (6)	14 (10–29)
Bypass	16 (2.6)	14	2	69 (59–77)	25 (4.8)	21 (13–32)
Amputation	23 (3.8)	17	6	71 (57–76)	31 (5.31)	27 (17–32)
EVAR	7 (1.2)	2	5	71 (49–87)	19.4 (7.07)	9 (7–31)
Embolectomy	10 (1.7)	9	1	77 (64–82)	14.5 (4.83)	8 (8–16)
Others	1 (0.2)	0	1	72	17 (0)	17
**General surgery**	**346 (57)**	**248**	**98**	**36(26–50)**	**6 (0.51)**	**3 (2–6)**
**Abscesses**	136					
Perianal Abscess	55 (9.1)	42	13	41 (32–48)	2.4 (0.24)	2 (1–3)
IVDU-related Abscess	32 (5.3)	16	15	34 (28–44)	3.1 (0.77)	2 (1–3)
Pilonidal Abscess	12 (2)	7	5	28 (21–37)	2 (0.33)	2 (1–2.5)
Hydradenitis abscess	2 (0.3)	2	0	41 (39–42)	2 (0)	2 (2–2)
Other abscess	35 (5.8)	21	14	40 (26–48)	2.8 (0.62)	2 (1–3)
**Laparoscopy**	**74**					
Laparoscopic appendectomy	43 (7.1)	23	20	26 (21–31)	3.1 (0.3)	3 (2–4)
Laparoscopic DUP repair	3 (0.5)	1	2	66 (62–76)	7 (4.04)	4 (3–15)
Laparoscopic Cholecystectomy	17 (2.8)	7	**10**	44 (34–59)	9.2 (2.84)	6 (4–9)
Laparoscopic exploration	7 (1.2)	1	**6**	23 (18–51)	6.9 (1.95)	6 (3–10)
Other laparoscopy	4 (0.7)	2	2	52 (39–65)	17.3 (5.85)	15 (8–26)
**Laparotomy**	**135**					
Laparoptomy appendicectomy	5 (0.8)	2	**3**	39 (37–57)	1 (0.46)	6 (3–7)
Open appendicectomy	45 (7.5)	33	12	27(23–34)	4.13 (0.81)	2 (2–4)
Open DUP repair	6 (1)	5	2	41 (35–72)	9 (3.06)	6 (4–8)
Obstruction	12 (2)	7	5	60 (38–73)	24.3 (5.73)	16 (7–37)
Other laparotomy	30 (5)	10	**20**	50 (37–70)	15.3 (3.52)	9 (5–15)
Hernia	14 (2.3)	12	2	42 (29–58)	5.9 (2.4)	2 (2–5.5)
**Other procedures**	24	13	8	42 (33–54)	9.8 (4.2)	5 (3–11)
**Overall**	**605**	**458**	**147**	**37 (25–52)**	**10 (0.6)**	**4 (2–11)**

N: number. M: male. F: female. SEM: standard error of mean. IQR: interquatile range. AAA: abdominal aortic aneurysm. EVAR: endovascular aneurysm repair. IVDU: intravenous drug usage. DUP: duodenal ulcer perforation.

### Length of stay

**Figure 5:**
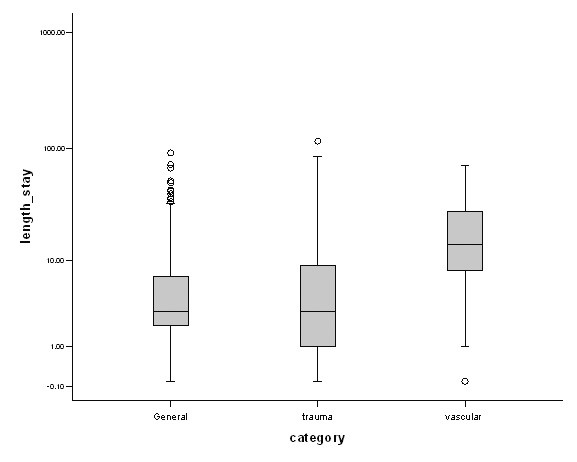
Median length of stay according to specialties. Results are expressed as median ± Interquartile range. Y-axis is log transformed.

**Figure 6:**
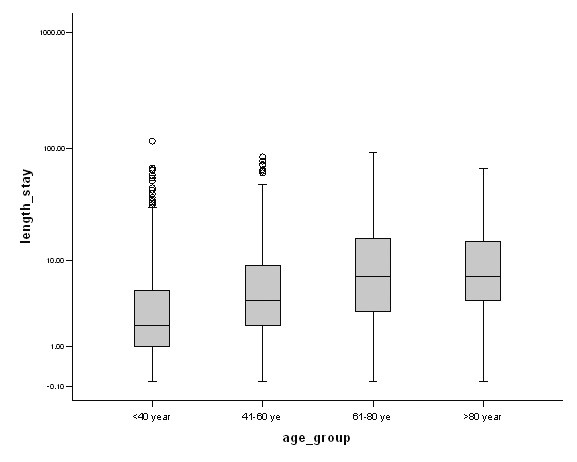
Median length of stay according to age groups. Results are expressed as median ± Interquartile range. Y-axis is log transformed.

The median length of stay was 4 (2–8) days. Vascular patients stayed in hospital significantly longer than trauma or general surgery patients (Kruskall Wallis, df = 2; Chi-square statistic = 106.8, P < 0.001; Figure 5), possibly because most vascular patients were elderly. Elderly patients (> 80 years) stayed 6 days longer in the hospital as compared to those below the age of 40 years (Kruskall Wallis, df = 3; Chi-square statistic = 129.7, P < 0.001; Figure 6). Patients who underwent surgery stayed a median of 4 days as compared to those without surgical intervention (3 days). It is worthwhile noting that laparoscopic appendicectomy patients stayed a median of 3 days as compared to 2 days for open appendicectomy. All abscesses stayed a median of 2 days in hospital.

## Discussion

### Data uniqueness

The strength of this contemporaneous data from one surgical department in the UK is prospective collection with complete follow-up, along with time of intervention. This allows us to get some estimates on the dynamic nature of emergency surgery, an aspect which is generally underestimated. Inevitably, a single centre data has limitations as to the generalizations which can be derived from them. In addition, there are other aspects of this work, which should be borne in mind. The Royal London Hospital serves a young, multiethnic, socio-economically deprived and diverse, largely immigrant population of the Tower Hamlets borough of London. This population has higher rates of accidental injuries than the rest of England. [5] It is, also, the site of the only Helicopter emergency medical service (HEMS) in London. [7,8] As a result, there is a higher intake of trauma patients, compared to the rest of the UK, who tend to be younger. This would explain lower median age of 41 (28–60) years as compared to other UK studies [46.9 (range 12–99) years in 1984 and 52.6 years in 1998. [4,9-12] Though only 6% of patients were brought in by the HEMS, they considerably impacted on the workload [8,9] as they were usually poly-trauma patients requiring muti-specialty care and investigational facilities as well as theatre space. Moreover, they also tended to stay in hospital longer despite a younger median age (Table 1).

The incidence of common general surgical diagnoses such as abscess, and non-specific abdominal pain, was similar to those reported by Stower et al [12] and Irvin [11]. The much higher proportion of trauma admissions (28%) compared to the 5.3% reported by Stower et al [12] and to the 2% reported by Bain et al [9], is in keeping with the prominent role of a designated major trauma center for London. Only 11% of patients were referred by General Practitioner (GP) and there were a high proportion of self-referral patients, and those brought in by ambulances (surface and air). Dookeran et al suggested that up to 41% of such admissions referred by General Practitioner were inappropriate [13] which could not be confirmed in our study.

### Timing of admissions and length of stay

Monday was peak day of admission with Saturdays experiencing a dip which is different from results of Stower et al who demonstrated that there was no change of admission rate over the week. [12] Possible explanation could be proximity to a large work-force of over a million extra individuals over the working week in the City of London, a major financial services hub. Additionally, as can be expected the peak time for admissions during the day was from mid-day to mid-night, and this fact, makes a case for appropriate resource allocation.

The overall median length of stay of surgical emergency patients was 4 (IQR 2–8) days. This was significantly shorter than the mean length of stay for both elective and emergency cases in all specialties in the UK (7.1 days) as reported by HES (Hospital Episodes Statistics) online in 2003–2004 and reflects the dynamic nature of emergency surgical admissions. [14] Older patients and vascular emergencies (also an older cohort) tended to stay longer possibly due to co-morbidities. [4,10,15,16] The discharge delays were often due lack of provision of social services and rehabilitation after discharge. [14,16,17] Polytrauma patients also tended to stay longer. It is interesting that despite RCTs showing a shortened stay for laparoscopic procedures over open procedures, this is not always borne out in day-to-day clinical practice, for procedures such as appendicectomy (median stay of 3 days for laparoscopic versus 2 days for open procedure). [18]

### Types of operation

The emergency operation rate (43.5%) was much higher compared to around 30.3% in other series. [9] This could be due to the high number of trauma patients and tertiary referral status or perhaps just a higher threshold for admitting patients, due to availability of investigations in ER. Incision and drainage of abscess (I&D), the commonest operation was performed in 22.5% of all operative cases. This again differs from all previous studies in which the commonest emergency procedure was appendicectomy (15% appendicectomy Dawson study, 11% in Stower study). [2-4,10,15] However the abscesses stayed for a median of two days (two overnight stays, the first of them being for waiting for a theatre slot). This may reflect the lower prioritization for emergency theatre space, given other demands in a major trauma center, where only one theatre is fully staffed for emergency general surgical operations. Better theatre and admission planning would be required. A current pilot scheme looks at discharge from A&E followed by same/next day admission for urgent surgery (such as I&D) on a planned emergency list. It is interesting to note that though the admission sex ratio (M:F) was 1.7:1, the ratio for operation was 3.1:1 (tables 1 and 2). This is due to male predominance for trauma patients (requiring a higher rate of operative interventions) and female predominance for non-specific abdominal pain, gall bladder and diverticular disease as well as gynaecological conditions; all of which had lower intervention rate. Thus, only operations such as diagnostic laparoscopies, other laparotomies (often diagnostic and for diverticular disease) and emergency laparoscopic cholecystectomy were more common for women.

### Training

A major trauma centre and tertiary teaching hospital offers an array of training opportunities for the trainee surgeon through exposure to a wide-range of poly-trauma and emergency surgical patients requiring urgent surgical decisions and frequent operative intervention and a rapid turnover. [19] With the introduction of European Working Time Directive (EWTD, an Europe-wide legislation leading to reduction of average working hours from 72 hours to 48 hours) [20] and consequent reduction in the exposure to elective surgery, the findings of this study suggest that trainees gain valuable experience from exposure to a broad spectrum of emergency surgery in a major trauma centre. This fact should be reflected in their training curriculum and log-book. Data such as these should also be considered for workforce planning during re-organisation and rationalisation of health services similar to those planned for London [6]

## Authors' contributions

TAMP collected and analysed data, BP, MW, ATA were instrumental in deciding the type of data to be collected and in formulating the study design and contributed critically at all meetings during the progress of this research, HMK conceived the idea, directed the research, analysed data and wrote the paper. All authors read and approved the final manuscript.
